# Multiple Sclerosis in a Patient With Prior West Nile Encephalitis

**DOI:** 10.7759/cureus.28935

**Published:** 2022-09-08

**Authors:** Brian Bui, Julianne Byun, Jamie Jacobs, Antonio K Liu

**Affiliations:** 1 Internal Medicine, Adventist Health System, Los Angeles, USA; 2 Internal Medicine, Loma Linda University School of Medicine, Loma Linda, USA; 3 Internal Medicine, Adventist Health White Memorial, Los Angeles, USA; 4 Neurology, Adventist Health White Memorial, Los Angeles, USA; 5 Neurology, Loma Linda University School of Medicine, Loma Linda, USA

**Keywords:** pathophysiology, inflammatory, induction, west nile encephalitis, multiple sclerosis

## Abstract

Multiple sclerosis is a neurodegenerative disease characterized by an inflammatory demyelination of the central nervous system. The degenerative disease has been linked to numerous viral infections, geographical locations, and genetic predisposition. One link that has not been fully established is the relationship between West Nile virus infection and its role in the initiation of multiple sclerosis. This case study provides further evidence that the proinflammatory neurological processes induced by the West Nile virus may lead to systemic demyelination of neuronal axons, ultimately causing multiple sclerosis.

## Introduction

Multiple sclerosis (MS) is a chronic, degenerative central nervous system (CNS) disease that is caused by an inflammatory demyelination of oligodendrocytic axons. The pathophysiology of MS involves the activation of autoreactive T-lymphocytes which induce inflammation and demyelination of CNS axons. Axonal loss causes CNS neurons to degenerate and accumulate into chronic plaques seen in the white matter of the brain. Although its etiology is not fully understood, activation and flares have largely been linked to genetic predisposition and can also commonly be triggered by viral infections. Previous studies have shown that there is an increased risk of developing MS in individuals with specific alleles in the major histocompatibility complex (MHC), specifically HLA-DRB1 [1]. The presence of alleles HLA-DRB1 04, 07, and 09 were associated with a reduced risk of developing MS while DRB1 15, 16, and 08 were associated with an increased risk of MS [2]. Furthermore, there have been studies showing strong associations between the Epstein-Barr virus (EBV) and MS [3]. We present a patient with encephalitis from West Nile virus (WNV) followed by the development of signs and symptoms that fulfilled the modified McDonald criteria for the diagnosis of MS four years later. WNV is a mosquito-borne virus that has the potential to progress to meningoencephalitis. Long-term neurological sequelae have been reported including demyelinating neuropathy, transverse myelitis, and symptoms similar to Guillain-Barre and neurodegenerative diseases. There are currently no known links between WNV and MS in the literature.

## Case presentation

The patient is a 20-year-old female with a past medical history of WNV meningoencephalitis diagnosed in 2017 with subsequent development of behavioral disinhibition consistent with Kluver-Bucy syndrome who presented to the Emergency Department for evaluation of recent onset left-sided upper extremity and lower extremity weakness. The patient stated that the weakness and paresthesia began approximately 5 to 7 days prior to admission, with symptoms worsening. Neurological examination presented with no focal motor deficits, but subjective paresthesia and decreased sensation specifically on the left upper extremity were noted. She had dysmetria and dysdiadochokinesia on the left upper and lower extremities. She was unable to walk unassisted. Upon admission, the patient had an MRI of the brain with contrast performed. MRI findings showed T2 hyperintensities located in the periventricular region, corpus callosum, right midbrain, and right brachium pontis. The cerebellum, pons, midbrain, and cerebral peduncles were noted to have normal signal intensity. The patient denied past history of weakness, paresthesia, urinary incontinence, transient vision loss, and other common sequelae of multiple sclerosis.

Medical records were reconciled from an outside-hospital facility and reviewed. By review, the patient had an extensive hospital course for WNV meningoencephalitis in 2017 with slow recovery over a period of 1 year. Lumbar puncture (LP) was performed and confirmed active WNV infection. CT Head 2017 showed no focal findings. Repeat LP was performed in 2019 to assess for recurrent infection on outpatient follow-up. Cerebral spinal fluid (CSF) showed WNV IgG Ab (2.15), WNV IgM Ab (< 0.90), positive oligoclonal bands, myelin basic protein (< 2.0 mcg/L), lymphocytes (82%), monocytes/macrophages (12%), RBC (121 cells/mm3), WBC (1 cell/mm3), negative culture, segmental (6%) (Table 1).

**Table 1:** Cerebral spinal fluid (CSF) analysis in 2019 with positive West Nile virus IgG and oligoclonal bands.

CSF white blood cells	1 cell/mm^3^
CSF red blood cells	121 cells/mm^3^
CSF lymphocytes	82%
CSF monocytes	12%
CSF myelin basic protein	< 2.0 mcg/L
CSF West Nile virus IgG antibody	2.15 (reference 1.29 or less)
CSF West Nile virus IgM antibody	<0.90
CSF oligoclonal bands	positive

**Figure 1:**
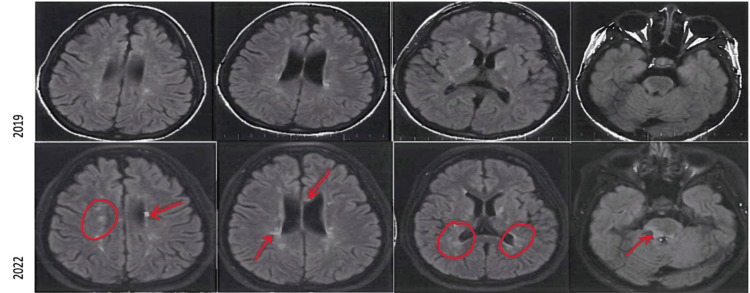
MRI FLAIR sequences from 2019 (above) and 2022 (below) showing increased white matter disease (indicated by red arrows and circles) at periventricular white matter areas, corpus callosum, and brainstem. FLAIR: Fluid-attenuated inversion recovery

**Figure 2:**
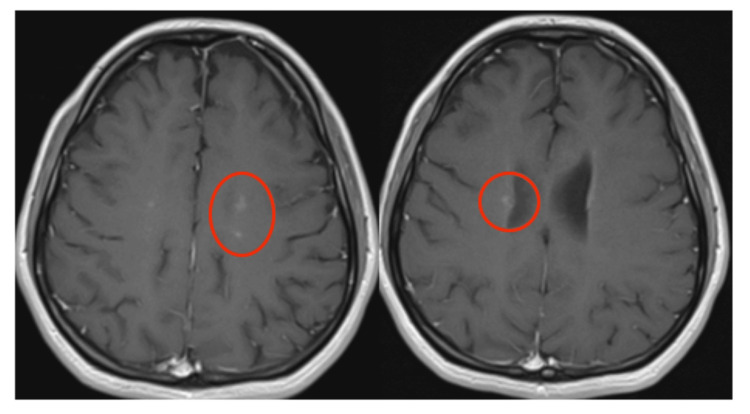
MRI head revealed enhancements (red circles) in post-contrast T1 sequence.

MRI Brain in 2019 showed multiple small foci of T2/FLAIR (fluid-attenuated inversion recovery) signal hyperintensity involving the bilateral cerebral and cerebellar white matter, bilateral basal ganglia, thalami, and brainstem. By utilizing the modified McDonald criteria for MS, the patient met the criteria for MS with one clinical attack, greater than two lesions with objective clinical evidence. MRI comparison from 2019 to 2022 showed new-onset white-matter lesions on FLAIR sequences (Figure 1) with enhancement on T1 post-contrast study (Figure 2), and oligoclonal bands were present on lumbar puncture in 2019.

The patient refused repeat LP during the recent hospitalization. The patient was started on 125 mg of methylprednisolone every 6 hours and monitored over a six-day hospital course. Throughout the remainder of her hospital course, the patient reported a significant improvement in her left-sided weakness and paresthesia.

## Discussion

WNV has been implicated in the induction of multiple auto-inflammatory neurological disease processes, but its link to MS has not been fully established.

In a 2020 study, research on the pathophysiology of how WNV could induce neurological disease was tested in rodent models [4]. It was determined that WNV may induce neuronal degeneration through the induction of pro-inflammatory cytokines, utilizing inflammatory pathways common to Alzheimer’s and Parkinson’s. Both WNV and neurodegenerative disorders induce the accumulation of misfolded proteins in neurons, causing neuronal dysfunction and subsequent apoptosis. Neuronal apoptosis activates microglial phagocytosis and astrocyte recruitment resulting in the accumulation of microglial nodules and decreased neurogenesis. Throughout this pathway, inflammatory cytokines are released, such as interferon‐gamma (IFN‐γ), which has neurotoxic effects and can induce neuronal apoptosis. The mechanisms by which WNV may cause neurodegenerative disorders provide insight into its mechanism of action for inducing MS. The pathways for MS and other neurodegenerative diseases are similar in mechanism. Inflammatory effects of WNV can ultimately lead to axonal loss and the accumulation of plaques in the white matter, inducing MS.

A separate mechanism of action was assessed in a 2021 study. One neurological manifestation of WNV is acute flaccid paralysis. WNV can invade the CSF and causes inflammatory damage in the anterior horn of the gray matter in the spinal cord. These damaged nerves can present similarly to poliomyelitis with monoparesis, asymmetric paraparesis, or quadriparesis. Damage to the spinal cord gray matter can rarely manifest as acute transverse myelitis. A WNV case in Massachusetts showed “radiographic evidence displaying enhancement in 3 contiguous spinal cord segments as well as CSF analysis consistent with West Nile neuro-invasive disease” [5]. This indicates that WNV induces longitudinally extensive transverse myelitis. WNV-induced inflammatory damage to the myelin of the spinal cord can subsequently cause demyelination. In 2016, a case study on WNV provided radiographic evidence of white matter disease within a span of two weeks [6]. Although WNV has a predilection for the brainstem, cerebellum, and the anterior horn cells of the spinal cord, it has the potential to invade different sites in the neural axis. If WNV invades the white matter of the brain, it can eventually cause demyelination of oligodendrocytes, precipitating MS.

## Conclusions

Multiple sclerosis is the demyelination of oligodendrocyte axons that has been associated with genetics, environment, and numerous viral infections that induce proinflammatory states. The EBV has long been the key virus associated with triggering MS, but in this study, there is evidence that WNV is a different virus that can be associated with the onset of MS. The mechanism of demyelination in white matter spaces induced by the WNV ultimately provides insight into the pathophysiology of how the WNV can cause multiple sclerosis. 
